# Liver Abscess in Children: A 10-year Single Centre Experience

**DOI:** 10.4103/1319-3767.80384

**Published:** 2011

**Authors:** Roohollah Salahi, Seyed M. Dehghani, Heshmatollah Salahi, Ali Bahador, Hamid R. Abbasy, Fatemeh Salahi

**Affiliations:** 1 Trauma Research Center, Iran, Shiraz, Sciences Medical of University Shiraz, Center Archrese, Shiraz, Iran; 2 Transplant Research Center, Shiraz, Iran; 3 Master of Organic Chemistry, Shiraz, Iran

**Keywords:** Children, clinical findings, complications, liver abscess, treatment

## Abstract

**Background/Aim:**

Although liver abscess is more prevalent in developing countries than in developed countries, there is scant data about the characteristics of pediatric liver abscess in our region. We aimed to analyze the characteristics of pediatric liver abscess in our region and compare these with those of developed countries.

**Materials and Methods:**

The clinical features, laboratory, imaging, microbiologic findings, management strategy, and final outcome were extracted from the patients’ records retrospectively.

**Results:**

There were 18 cases of liver abscess including 16 pyogenic liver abscess, one amebic liver abscess and one *candida* liver abscess. Fever and abdominal pain were the most common clinical findings and leukocytosis was the most common laboratory finding. The most predisposing factors of liver abscesswere immune deficiency, minor thalassemia. Origin of liver abscess was appendicitis in two patients, the rest were considered as cryptogenic. While one patient was treated with antibiotics alone, five cases were taken for open drainage, and 12 cases were treated with percutaneous aspiration. Percutaneous aspiration failed in two patients who were later ttaken for open drainage, with an overall mortality rate of 5.5%. Conclusion: The overall characteristics of liver abscess in children in our society are not so different from developed countries. However, in contradiction to cases reported in developed countries, most cases of liver abscess were seen in healthy patients in our centre. Moreover, liver abscess was reported in our patients at a younger age and was more commonly seen in male children. Mortality rate was similar to that of developed countries.

Liver abscess (LA) is a serious infectious disease, which is responsible for hospitalization and mortality worldwide.[1] LA is rare with an incidence of 25 per 100,000 admissions in USA; however, in studies conducted in developing nations it was reported in 1 out of 140 admissions.[2] Although it is supposed that in developing countries as in Iran, infectious diseases like LA are more prevalent than in developed countries, there is little data about the characteristics of pediatric liver abscess in our region, and management of our patients has been based on data provided through studies conducted and published in developed countries. The aim of this study has been to identify the characteristics of LA in children referred over the last decade to our center and to review their clinical features, predisposing factors, management, and mortality and compare them with the results in developed countries.

## MATERIALS AND METHODS

The records of all patients less than 18 years who were admitted in pediatric wards at Nemazee Hospital, referral center in South of Iran, with diagnosis of LA between 1998 and 2008 were reviewed. Age, sex, symptoms and their duration, clinical signs at presentation and any underlying predisposing factors were noted. Results of the diagnostic work-ups including ultrasonography (US) findings and organisms isolated, management including details of antibiotic therapy and drainage procedure were all recorded. In-hospital mortality was defined as mortality that occurred within the same hospital. Length of hospital stay was defined as total days since admission to discharge. There was a two-year follow-up on all patients to check for any recurrences or complications. The patients were subsequently followed up in the clinic. Statistical package for social sciences (SPSS, version 15.0; Chicago, IL, USA) was used for data analysis.

## RESULTS

There were 18 cases (11 boys and 7 girls) including PLA (*n:*16), amebic liver abscess (*n:*1), and *candida* LA (*n:*1). The median age was eight years (range, 50 days-17 years). The predisposing factors of LA were seen in six cases including immune system diseases with one case of severe combined immunodeficiency (SCID) and one case of chronic granulomatous disease (CGD), minor thalassemia (*n:*2), acute lymphocytic leukemia (*n:*1), and kala-azar (*n:*1). Median time of delay between the beginning of the symptoms and the diagnosis was 13 days (range, 3-90 days). Origin of LA was appendicitis in two cases (11.1%) and cryptogenic (i.e. without a definite cause) in sixteen cases (88.9%).

Fever and abdominal pain were seen in 94.4% and 88.9% respectively. Tenderness in the right hypochondrium was present in all cases [Table 1].

**Table 1:** Demographic data, clinical characteristics, imaging findings (location, size, number of liver abscess), type of management, and outcome of patients

No.	Type of abscess	Time of presentation	Sex/age (years)	Symptoms	Associated conditions	Imaging findings	Treatment	Complication
1	Pyogenic	3-90 days	M/13	Fever, abdominal pain, vomiting, weight loss	Thalassemia	Single, left lobe	Percutaneous aspiration	
2	Pyogenic	3-90 days	F/5months	Fever, abdominal pain, vomiting, weight loss		Single, left lobe	Percutaneous aspiration	
3	Pyogenic	3-90 days	M/16	Fever, abdominal pain, weight loss	CGD	Two, left lobe	Laparotomy	Wound infection recurrence
4	Pyogenic	3-90 days	F/6	Fever, abdominal pain, vomiting	Appendicitis	Multiple, right and left lobe	Laparotomy	Pericardial effusiom rupture to peritoneum
5	Pyogenic	3-90 days	M/15	Fever, chills, anorexia, abdominal pain	Thalassemia	Two, right lobe	Percutaneous aspiration	
6	Pyogenic	3-90 days	F/13	Fever, abdominal pain chills, anorexia,		Two, right lobe	Laparotomy	Pleural effusion
7	Pyogenic	3-90 days	M/17	Fever, abdominal pain, weight loss, anorexia		Single, right lobe	Antibiotic	
8	Pyogenic	3-90 days	M/11	Fever, abdminal pain, chills	ALL	Single, left lobe	Percutaneous aspiration	Pleural effusion
9	Pyogenic	3-90 days	F/5	Fever, abdominal pain, anorexia	Kala-azar	Single, right lobe	Percutaneous aspiration	
10	Pyogenic	3-90 days	M/3	Fever, anorexia, weight loss		Single, right lobe	Percutaneous aspiration	
11	Pyogenic	3-90 days	M/5	Fever, abdominal pain, vomiting		Single, right lobe	Percutaneous aspiration	
12	Pyogenic	3-90 days	F/4	Fever, abdominal pain anorexia, vomiting	Appendicitis	Two, right lobe	Laparotomy	Rupture to peritoneum
13	Pyogenic	3-90 days	F/8	Fever, abdominal pain chills, vomiting		Single, right lobe	Percutaneous aspiration	
14	Pyogenic	3-90 days	M/15	Fever, abdominal pain, chills		Single, right lobe	Percutaneous aspiration	Pleural effusion
15	Pyogenic	3-90 days	M/12	Fever, abdominal pain, chills, vomiting		Single, right lobe	Percutaneous aspiration	
16	Pyogenic	3-90 days	M/16	Fever, abdominal pain, weight loss		Single, left lobe	Percutaneous aspiration	Pleural effusion recurrence
17	Ameobic	30 days	M/3	Abdominal pain, anorexia		Single right lobe	Single right lobe	
18	*Candida*	12 days	F/50 days	Fever, vomiting	SCID	Multiple, right and left lobe	Laparotomy	Death due to sepsis

CGD: chronic granulomatous disease; M: Male; F: Female, SCID: severe combined immunodeficiency; ALL: acute lymphoblastic leukemia

US showed single lesion in 12 cases, two abscesses in four cases, and multiple lesions in two cases. LA was seen in the right hepatic lobe in 11 cases, left lobe in five cases, and bilateral in two cases. The most common abnormal laboratory data included leukocytosis (94.4%), and anemia (88.9%). Laboratory data are shown in Table 2. Pus culture was positive in 50% of PLA (8 out of 16), including staphylococcus species in six cases (*Staphylococcus auerus* in five patients and staphylococcus coagulase negative in one case), Klebsiella in one case and Enterobacter in one case. Culture was negative in the rest of 10 patients (55.5%). Blood culture was positive only in one case of PLA which grew coagulase negative *Staphylococcus aureus*.

**Table 2:** Abnormal laboratory data findings in pediatric liver abscess

	WBC	Hemoglobin*	Globulin*	Platelet	Albumin*	AST#	Bilirubin*	ALT#
Normal	>10000	<11	>3.5	>350000	<3.5	>40	>1.4	>40
Mean	28823	9.8	3.8	397705	3.6	30.3	1.1	19.1
Abnormal%	94.1	82.3	68.7	58.8	47	25	23.5	12.5

* :mg/dl

# :IU/L, WBC: white blood cell, ALT: alanine transaminase, AST: aspartate transaminase

A combination of ampicillin plus aminoglycoside plus metronidazole were used in patients with PLA for two weeks intravenously and continued for four weeks orally till about three years ago; but because of growing antibiotic resistance, we now use vancomycin plus imipenem, as empirical therapy, and then change antibiotics according to the results of culture. Out of 16 cases of PLA, one patient was treated with antibiotics alone. Percutaneous aspiration was done in 11 cases (68.75%) of whom two patients (cases 2, 11) did not show much improvement, and had to undergo open drainage. The most common complications included right sided pleural effusion (*n:*4), which resolved without any treatment, and recurrence of LA which happened in one patient after two months (case 3) and in the other patient (case 16), two years after percutaneous aspiration. These patients underwent open drainage with good clinical response. In these patients, immunological studies such as absolute lymphocyte and neutrophil counts, serum immunoglobulins, and nitroblue-tetrazolium test were normal and there were no underlying predisposing factors. Rest of the four patients of pyogenic group (case 3, 4, 6, and 12) underwent open drainage at presentation due to rupture of LA into peritoneum (*n:*1), rupture of appendicitis into peritoneum (*n:*2) and CGD (*n:*1).

Amebic liver abscess (ALA) was seen in a three-year-old boy who was referred due to fever and anorexia. The diagnosis of ALA was established by US evidence of a single hypoechoic lesion in right lobe of the liver with positive indirect hemagglutination test (>1:250). He was treated with intravenous metronidazole, followed by percutaneous drainage due to non-improvement in clinical situation. Liver aspirate showed eosinophil cells and charcot leidon body in favor of ALA.

Diffuse candidiasis occurred in a 50 days infant with SCID. US showed numerous target lesions in liver in favor of candidiasis and urine culture grew *candida*. Abscess was multiple and located in both lobes. Despite open drainage and broad spectrum antifungal treatment, he did not respond to therapy and died two weeks after admission due to septic shock and multiorgan failure.

## DISCUSSION

PLA has been described to be rare in infancy and childhood, but it still remains a major cause of high mortality in children.[1] In developed countries, LA occurred in children with immune system disease especially CGD.[3] However in our center like other developed countries,[4] the majority of patients were healthy. This might be due to high rate of environmental infection in developing countries.

The median age at presentation was eight years which is similar to other developing countries like Taiwan[5] and Brazil.[2] However, in United Kingdom, median[6] age at diagnosis was 10 years. These findings suggest an earlier age trend of LA in patients in developing countries compared to that in developed countries which may be due to earlier contact with infection. Despite studies from both developing and developed countries that reported no difference between girls and boys,[5–7] in our series boys were involved more than girls.

Staphylococcus is the most common cause of LA in children,[6] which is similar to our study. Pus and blood culture was positive for 55% and 58% of patients.[8] The most common organism identified was *Klebsiella* species followed by *Escherichia coli*.[8] Anerobic organisms are increasingly being reported as a causative agent in PLA in children.[3,9] The low level of positive blood culture in our study could partly be due to prior antibiotic therapy before admission and non availability of facility for anerobic culture in most cases.

There were no differences about symptoms and signs of LA[10] and also initial use of US to diagnose LA.[6] Increased alkaline phosphatase and low albumin have been reported as the most common abnormal laboratory findings;[11] however, elevated levels of billirubin and aminotransferase and leukocytosis are also common.[12] In our cases leukocytosis was the most common abnormal finding, followed by anemia (Hb<11 mg/dl).

Controversy exists on whether to treat LA with medical treatment alone or to use percutaneous or surgical drainage together with antibiotic therapy. A course of six weeks antibiotic therapy alone, including two weeks intravenously, followed by four weeks orally is recommended, when multiple abscesses are too small (less than 2 cm), to be drained percutaneously.[13] Percutaneous drainages are recommended when the abscess is large or seems to rupture on US, or does not respond to antibiotic therapy after 72 h.[10,13] The indications of open laporotomy include nonresponse to percutaneous drainage together with antibiotic therapy, or when the pus is thick, in multiloculated abscess[10,13,14] and LA in CGD patients[15] because in these patients the response of abscesses to drainage procedures and antibiotic therapy is poor and high rate of recurrence was reported with these methods.[16] However, in our center three pediatric surgeons have the same protocol as in other developing and developed countries to manage liver abscess. We treated pyogenic liver abscess in children with ampicillin plus aminoglycoside plus metronidazole empirically till about three years ago but due to growing antibiotic resistance we use vancomycin plus imipenem now as empirical therapy, and then change antibiotics according to the results of culture. If the abscess is assessable we drained it percutaneously under sonographical guidance. However, the indication of laporotomy includes the following: 1. Multiseptated abscess 2. LA in CGD patients 3. Associated disease like appendicitis 4. Rupture of liver abscess 5. When response to percutaneous drainage together with antibiotic therapy is not favorable.

Uncomplicated ALA is treated medically by metronidazole. Percutaneous aspiration is also added if LA is larger than 6 cm or shows features of rupture on US, or nonresponse to medical therapy, or if the patient is septicemic.[13,17,18] Failure rate with percutaneous drainage was reported in 20% of cases in recent studies.[19] In our center, percutaneous drainage was the most common type of treatment in the management of PLA. The high rate of failed percutaneous drainage (18.18%) in our center, may be due to the fact that only needle aspiration was done and no percutaneous catheter drainage was inserted which might be a better procedure. Mortality of LA has decreased significantly during recent years from 36% in 1998[11] to 5.5% in 2007.[13] In our center mortality was seen in one patient (5.5%) which was a case of SCID with *candida* liver abscess.

## References

[1] Vanni LA, Lopez PB, Porto SO (1978). Solitary pyogenic liver abscess in children. Am J Dis Child.

[2] Ferreira MA, Pereira FE, Musso C, Dettogni RV (1997). Pyogenic liver abscess in children: Some observations in the Espírito Santo State, Brazil. Arq Gastroenterol.

[3] Pineiro-Carrero VM, Andres JM (1989). Morbidity and mortality in children with pyogenic liver abscess. Am J Dis Child.

[4] Mehta RB, Parija SC, Chetty DV, Smile RR (1986). Management of 240 cases of liver abscess. Int Surg.

[5] Tsai CC, Chung JH, Ko SF, Liu PM, Su CT, Li WC (2003). Liver abscess in children: A single institutional experience in southern Taiwan. Acta Paediatr Taiwan.

[6] Muorah M, Hinds R, Verma A, Yu D, Samyn M, Mieli-Vergani G (2006). Liver abscesses in children: A single center experience in the developed world. J Pediatr Gastroenterol Nutr.

[7] Chou FF, Sheen-Chen SM, Chen YS, Chen MC (1997). Single and multiple pyogenic liver abscesses: Clinical course, etiology, and results of treatment. World J Surg.

[8] Wong W, Wong B, Kin Hui C, Ng M, Cuen Lai K, Kuen Tso W (2002). Pyogenic liver abscess: Retrospective analysis of 80 cases over a 10-year period. J Gastroenterol Hepatol.

[9] Barnes PF, De Cock KM, Reynolds TN, Ralls PW (1987). A comparison of amoebic and pyogenic abscess of the liver. Medicine (Baltimore).

[10] Kumar A, Srinivasan S, Sharma AK (1998). Pyogenic liver abscess in children-South Indian experiences. J Pediatr Surg.

[11] Donovan AJ, Yellin AE, Ralls PW (1991). Hepatic abscess. World J Surg.

[12] Israeli R, Jule JR, Hom J (2009). Pediatric pyogenic liver abscess. Pediatric Emergency Care.

[13] Bari S, Sheikh KA, Malik AA, Wani RA, Naqash SH (2007). Percutaneous aspiration versus open drainage of liver abscess in children. Pediatr Surg Int.

[14] Kaplan SL (1979). Pyogenic liver abscess in normal children with fever of unknown origin. Paediatrics.

[15] Chen LE, Minkes RK, Shackelford PG, Strasberg SM, Kuo EY, Langer JC (2003). Cut it out: Managing hepatic abscesses in patients with chronic granulomatous disease. J Pediatr Surg.

[16] Lublin M, Bartlett DL, Danforth DN, Kauffman H, Gallin JI, Malech HL (2002). Hepatic abscess in patients with chronic granulomatous disease. Ann Surg.

[17] Kapoor OP, Kapoor OP (1979). Multiple amoebic liver abscesses. Amoebic liver abscess.

[18] Moazam F, Nazir Z (1998). Amoebic liver abscess: Spare the knife save the child. J Pediatric Surg.

[19] Balint TD, Bailey BM, Mendelson KG, Pofahl W (2001). Hepatic abscess: Current concepts in diagnosis and treatment. Curr Surg.

